# Naturally occurring protease inhibitor resistance mutations and their frequencies in HIV proviral sequences of drug-naïve sex workers in Nairobi, Kenya

**DOI:** 10.1186/1742-4690-11-S1-P133

**Published:** 2014-01-07

**Authors:** Raghavan Sampathkumar, Elnaz Shadabi, David La, John Ho, Binhua Liang, Joshua Kimani, Terry B Ball, Francis A Plummer, Ma Luo

**Affiliations:** 1 Department of Medical Microbiology, University of Manitoba, Winnipeg, Manitoba, Canada; 2 Department of Medical Microbiology, University of Nairobi, Nairobi, Kenya; 3 National Microbiology Laboratory, Public Health Agency of Canada, Winnipeg, Manitoba, Canada

## 

Sub-Saharan Africa accounts for 69% of the people living with HIV globally. An estimated 1,600,000 Kenyans are living with HIV-1. Antiretroviral therapy (ART) has saved 9 million life-years in Sub-Saharan Africa. However, drug resistance mutations reduce the effectiveness of ART, and need to be monitored for effective ART. Naturally occurring primary antiretroviral drug resistance mutations have not been well analyzed in ART naïve HIV+ patients in Kenya. Here we have examined protease inhibitor (PI) resistance mutations in ART naïve HIV-1 seropositive women in Pumwani sex worker cohort established in Nairobi, Kenya, wherein HIV-1 infection is predominantly caused by subtypes A and D viruses. We have analyzed consensus sequences of HIV protease from 109 drug-naïve patients, as a part of HIV-1 whole-genome sequencing using 454 sequencing methodology. Analysis using HIVdb program revealed a prevalence of 22% (24/109) PI resistance mutations among the study subjects. D30N (3.7%), M46I (0.9%) and V82F (0.9%) are the major mutations observed. D30N mutation is known to confer high-level resistance to nelfinavir. M46I and V82F confer resistance to indinavir, lopinavir, fosamprenavir and nelfinavir. In addition, many minor mutations were found at seven different drug resistance sites. It is important to study the implications of these mutations to the effectiveness of specific PI drug treatment. This study provides valuable data pertaining to primary drug resistance in Kenyan HIV-1 infected patients before ART became available.

